# Bovine DDX3X Restrains Bovine SP110c-Mediated Activation of Inflammasome in Macrophages

**DOI:** 10.3390/ani14111650

**Published:** 2024-05-31

**Authors:** Zhunan Li, Jing Han, Jiayi Jing, Ajiao Fan, Yong Zhang, Yuanpeng Gao

**Affiliations:** Key Laboratory of Animal Biotechnology of the Ministry of Agriculture, College of Veterinary Medicine, Northwest A&F University, Yangling 712100, China; lizhunan2020@163.com (Z.L.); hanjing1995@yeah.net (J.H.); jingjiayi1998@163.com (J.J.); fanajiao2021@163.com (A.F.)

**Keywords:** bovine SP110c, bovine DDX3X, macrophages, NLRP3 inflammasome, pyroptosis

## Abstract

**Simple Summary:**

As a crucial component of the innate immune system, inflammasomes are activated by cellular infection or stress, which leads to the secretion of proinflammatory cytokines IL-1β and IL-18. In order to avert harmful inflammatory responses and maintain immune homeostasis, the precise control mechanisms of inflammasome activation need to be further defined. Previously, researchers have characterized the bovine SP110c isoform (bSP110c) as a new trigger of inflammasome activation in macrophages stimulated with bacterial lipopolysaccharide; however, the exact molecular mechanism for negative regulation of bSP110c-induced inflammasome remains unknown. In this study, the researchers demonstrated that bovine DDX3X is a novel bSP110c binding protein that restricts the bSP110c-mediated inflammatory response through the negative regulation of the NLRP3 inflammasome. These findings support a new cooperative mechanism between bSP110c and bovine DDX3X to balance inflammasome activation and advance our understanding of the function of the SP110c protein in the bovine innate immune system.

**Abstract:**

The inflammasome is a vital part of the host’s innate immunity activated by cellular infection or stress. Our previous research identified the bovine SP110c isoform (bSP110c) as a novel activator of the inflammasome that promoted the secretion of proinflammatory cytokines IL-1β and IL-18 in macrophages infected with *Listeria monocytogenes* or stimulated with lipopolysaccharide (LPS). However, the exact molecular mechanism for inhibiting bSP110c-induced inflammasome activation requires further clarification. Here, the researchers identified bovine DDX3X (bDDX3X) as an NLRP3-associated protein and an inhibitor of the bSP110c-induced inflammasome in the human THP1 macrophage cell line. Immunoprecipitation showed that bDDX3X interacted with the bSP110c CARD domain via its helicase domain. The co-expression of bSP110c and bDDX3X in THP1 macrophages significantly prevented the bSP110c-induced activation of inflammasomes. In addition, both bDDX3X and bSP110c interacted with bovine NLRP3 (bNLRP3), and bDDX3X enhanced the interaction between bSP110c and bNLRP3. The expression of bDDX3X in nigericin-stimulated THP1 macrophages significantly suppressed NLRP3 inflammasome activation, ASC speck formation, and pyroptosis. These findings demonstrate that bDDX3X negatively regulates the bSP110c-mediated inflammatory response by restricting the activation of the NLRP3 inflammasome. This discovery unveils a novel regulatory mechanism involving bDDX3X and bSP110c in coordinating inflammasome activation and subsequent cell-fate decisions in LPS-treated macrophages and, in turn, constitutes a step forward toward the implementation of marker-assisted selection in breeding programs aimed at utilizing cattle’s immune defenses.

## 1. Introduction

Inflammasomes are complex cytoplasmic assemblies of multiple proteins that play a vital role in the innate immune system by activating caspase-1 [1,2]. Caspase-1 activation enhances the maturation and secretion of inflammatory cytokines IL-1β and IL-18. Moreover, caspase-1 cuts gasdermin D (GSDMD), resulting in an extremely inflammatory type of cell death called pyroptosis [3,4,5]. Pyroptosis is identified by the swelling of the cytoplasm and the rupturing of the cell membrane. It shares similarities with necroptosis, except that the latter is independent of caspase [6,7,8,9]. Several inflammasomes have been identified. The name of each is determined by its NLR or ALR protein framework. Currently, the NLRP3 inflammasome is the most thoroughly studied. It consists of three parts: the NLRP3 sensor protein (nucleotide-binding domain (NBD)-containing protein and leucine-rich repeat (LRR)-containing protein), the ASC adaptor protein (apoptotic speck protein containing a caspase recruitment domain), and the caspase-1 effector protein [10,11,12]. NLRP3 has been detected in myeloid immune cells like neutrophils, monocytes, and dendritic cells and can be activated by microbial infections, endogenous damage signals, and pore-forming toxins [13,14,15].

The *SP110* gene, also known as the *intracellular pathogen resistance 1 (Ipr1)* gene in mice, was first discovered at the *super-susceptibility to tuberculosis 1 (sst1)* locus on chromosome 1, where it plays a vital role in the host’s innate immune reaction to infections [16,17,18]. Following infection with intracellular pathogens, the lungs of B6.Sst1^S^ congenic mice lacking *SP110* exhibited a significant increase in IL-1β levels [19,20]. Our previous research demonstrated that the bovine SP110c isoform (bSP110c) (GenBank, Accession No. AGT15802.1) is essential for an effective innate immune response. Bovine SP110c is a specific protein isoform translated from the bovine *SP110* gene. Compared to mouse SP110, bSP110c lacks the long carboxy-terminal structure, including the SAND domain [21,22,23]. The increased activation of the inflammasome, as well as the greater secretion of IL-1β and IL-18, occurred when bSP110c was expressed in THP1 macrophages infected with *Listeria monocytogenes* (*L. monocytogenes*) or primed with LPS. However, the mechanism that inhibits the bSP110c activation of inflammasomes is not well understood.

To identify the potential regulator of bSP110c-induced inflammasome activation, a pull-down assay was performed to screen for proteins from whole-cell lysates of bovine peripheral blood leukocytes that may interact with bSP110c. The proteins were identified by liquid chromatography coupled with tandem mass spectrometry (LC-MS/MS) analysis [24]. A possible protein candidate, known as bovine DDX3X (bDDX3X), was identified. DDX3X belongs to the DEAD/H-box RNA helicase family, with a helicase domain that is highly conserved and includes the DEAD (Asp-Glu-Ala-Asp)-box amino acid motif [25]. DDX3X is implicated in various diseases, including microbial infections, inflammation, intellectual disabilities, and cancer [26]. Mice without DDX3X show alterations in hematopoiesis and the types of white blood cells found in the bone marrow and spleen, making them much more vulnerable to *L. monocytogenes* infection [27]. In mouse macrophages, DDX3X is a major contributor to inflammasome complex assembly and interacts with NLRP3 to drive inflammasome activation [28,29,30].

This research shows that bDDX3X functions as a suppressor of the bSP110c-triggered inflammatory reaction by limiting the activation of NLRP3 inflammasomes. The discovery provides a fresh outlook on the strict control of the inflammasome by bSP110c working together with bDDX3X and provides a theoretical foundation for marker-assisted selection in developing disease-resistant cattle through biotechnological breeding.

## 2. Materials and Methods

### 2.1. Ethics Statement

The Animal Care and Use Committee of Northwest A&F University, China, approved the blood sampling procedure (Approval number NWLA-2020-058). Every effort was made to reduce the pain and discomfort experienced by the animals.

### 2.2. Reagents

Sigma-Aldrich (Burlington, MA, USA) provided PMA (phorbol 12-myristate 13-acetate) and LPS extracted from *Escherichia coli* (O55:B5). Red blood cell lysis buffer, DAPI, 4% paraformaldehyde solution, and blocking buffer for immunostaining were obtained from Beyotime Biotechnology (Shanghai, China). Nigericin was acquired from Solarbio (Beijing, China). For sterile CPD anticoagulant, a 100 mL solution contains trisodium citrate (2.63 g), citric acid (0.72 g), sodium dihydrogen phosphate (0.222 g), and glucose (2.55 g). 

### 2.3. Bovine Peripheral Blood Leukocyte Isolation

Jugular vein blood (200 mL) was aseptically collected from a 12–18-month-old female Holstein cow (Holstein–Friesian). The blood was stored at 4 °C in 50 mL sterile tubes pre-filled with CPD anticoagulant at a ratio of 15 mL per 100 mL of blood mixture. Subsequent operations were carried out on an ultra-clean bench. Blood samples were prepared by gently mixing anticoagulated blood with sterile PBS (calcium and magnesium ion-free) in a 1:1 ratio.

Bovine peripheral blood leukocytes were isolated using the peripheral blood lymphocyte isolation kit (HaoYang, Tianjin, China, LTS1077-1) [31]. Separation solution (ficoll configuration) and blood samples were added to a 15 mL sterile tube at a 5:4 ratio. First, 3 mL of separation solution was added, followed by the slow addition of 2 mL of blood sample along the tube wall to maintain layer separation. The tubes were then centrifuged at room temperature for 30 min using a horizontal rotor centrifuge at 400 *g*. After centrifugation, the tubes were separated into four layers: the top plasma layer, the second milky white lymphocyte layer, the third transparent separating fluid layer, and the bottom red blood cell layer. The plasma layer was carefully removed using a pipette, and the cell suspension in the lymphocyte layer was gently collected into a 15 mL sterile centrifuge tube. An equal volume of sterile PBS was added to the cell suspension, gently mixed, and centrifuged at room temperature for 15 min at 400 *g*. The cell pellet was collected, and 2 mL of red blood cell lysis buffer was added and incubated for two minutes. Subsequently, 5 mL of sterile PBS was added to the tubes, which were then centrifuged at 200 *g* for 15 min at room temperature. The cell pellets were retained as bovine peripheral blood leukocytes for further use.

### 2.4. Plasmid Construction

Total bovine peripheral blood leukocyte RNA was purified using TRIzol (Takara, Dalian, China) and reverse-transcribed with the HiScript II RT reagent kit (Vazyme Biotech, Nanjing, China). Bovine *SP110c* (KC858865.1), *DDX3X* (NM_001192962.1), *NLRP3* (NM_001102219.1), and *ASC* (NM_174730.2) were obtained through PCR amplification of cDNA from bovine peripheral blood leukocytes. Plasmids encoding bovine HA-tagged bNLRP3, HA-tagged bASC, HA-tagged bDDX3X, FLAG-tagged bDDX3X, and FLAG-tagged bSP110c were constructed using pcDNA3.1(+) (Invitrogen, Carlsbad, CA, USA). Truncated mutants of bSP110c and bDDX3X were generated by PCR-based mutation and amplification of wild-type bSP110c or bDDX3X expression vectors. Constructs of HA-tagged bDDX3X-ΔN-term (residues 179–661), bDDX3X-ΔC-term (residues 1–574), bDDX3X-helicase (residues 179–574), and FLAG-tagged bSP110c-C-term (residues 105–496) were created using the same vector background.

### 2.5. Cell Culture

PMA-activated human THP-1 macrophages (ATCC, Rockville, USA) were grown in RPMI-1640 (Procell, Wuhan, China, PM150116) with 10% (*v*/*v*) FCS (ExCell Bio, Taicang, China) and 0.05 mM β-mercaptoethanol (Solarbio, Beijing, China). HEK293T (ATCC, Rockville, USA) cells were cultivated in DMEM (Procell, Wuhan, China, PM150210) containing 10% (*v*/*v*) FCS (ExCell Bio, Taicang, China) and 100 mM sodium pyruvate (Solarbio, Beijing, China). All cells were cultured in a 37 °C incubator with 5% carbon dioxide.

### 2.6. Cell Stimulation and Transient Transfection

THP-1 and HEK293T cells were transfected with Lipofectamine 3000 (Invitrogen, Carlsbad, CA, USA) according to the manufacturer’s instructions. THP-1 cells were stimulated with 100 ng/mL PMA for 24 h. To study the activation of canonical NLRP3 inflammasomes, THP-1 cells were first primed with 100 ng/mL LPS for 4 h and then treated with 20 μM nigericin for 30–45 min.

### 2.7. Cell Viability Assay

The Cell Titer-Lumi™ Plus II luminescent cell viability assay kit was used following the instructions provided by the manufacturer (Beyotime Biotechnology, Shanghai, China).

### 2.8. Immunofluorescence Microscopy

THP-1 cells stimulated by PMA were seeded onto coverslips in 24-well plates and subsequently transfected with the expression plasmids indicated in the figure legends. Cells were transfected for a duration of 36 h, with an initial pretreatment of LPS for 4 h, followed by stimulation with nigericin for 45 min. The cells were collected by centrifugation and fixed with 4% paraformaldehyde at room temperature for 30 min. Afterward, the cells were blocked and made permeable in the buffer for blocking (Beyotime Biotechnology, Shanghai, China) for two hours. The cells were then exposed to the following antibodies for two hours at room temperature: anti-ASC (Proteintech, Wuhan, China, 10500-1-AP; diluted 1:100) and anti-NLRP3 (Proteintech, Wuhan, China, 68102-1-lg; diluted 1:100). Alexa Fluor 488-labeled goat anti-mouse IgG (Beyotime Biotechnology, Shanghai, China, A0516; diluted 1:1000) and Cy3-labeled goat anti-rabbit IgG (Beyotime Biotechnology, Shanghai, China, A0516; diluted 1:1000) were the secondary antibodies employed in the experiment. Cells were additionally stained with DAPI for nuclear visualization, and images were acquired with an FV3000 (Olympus Corporation, Tokyo, Japan) confocal microscope. The confocal images were processed using the manufacturer’s software (FV31S-SW, Version: 2.3.1.163).

### 2.9. Immunoblotting

After collecting the cells, they were promptly rinsed with PBS and subsequently lysed in a lysis buffer containing protease inhibitors and 5% NP-40. The samples were boiled in a loading buffer with the addition of SDS and β-mercaptoethanol for 10 min. Proteins were separated by SDS-PAGE on 10% or 15% gels and transferred to PVDF membranes. Membranes were blocked using 5% skim milk for 2 h at room temperature and then treated with primary antibodies and secondary antibodies linked to horseradish peroxidase (HRP) for 2 h each at room temperature. The antibodies purchased from Cell Signaling Technology (Boston, MA, USA) were anti-CASP1 (D7F10), anti-cleaved-IL-1β (Asp116), anti-GSDMD (E8G3F), anti-PARP (46D11), anti-Flag (D6W5B), and anti-HA (C29F4). Antibodies against HA (HT301-01), GAPDH (HC301-01), and β-tubulin (HC101-01) were acquired from TransGen Biotech (Beijing, China). Proteintech (Wuhan, China) provided antibodies against rabbit IgG (SA00001-2) and mouse IgG (SA00001-1). Protein bands were visualized using a multicolor fluorescence imaging system (Bio-Rad, Hercules, CA, USA, ChemiDocMP).

### 2.10. Enzyme-Linked Immunosorbent Assays (ELISAs)

After being stimulated, THP1 cell culture supernatants from cells that were transfected were tested for human IL-1β (Proteintech, Wuhan, China, KE00021) and IL-18 (Proteintech, Wuhan, China, KE00193) following the guidelines provided by the manufacturer. Cytokine concentrations were determined using a standard curve.

### 2.11. Co-Immunoprecipitation (Co-IP)

To perform co-IP, 293T cells were collected and lysed on ice in IP lysis buffer plus PMSF. Following the immunoprecipitation kit instructions, protein A + G agarose gel beads (Proteintech, Wuhan, China) were used for the immunoprecipitation procedure. The lysates from 293T cells were mixed with 4 μg of the specified antibody and incubated overnight at 4 °C on a rotating platform. Afterward, protein A + G agarose beads were added, and the mixture was then rotated for another 4 h at 4 °C. The agarose beads were centrifuged to collect the immunoprecipitated proteins and rinsed five times with ice-cold IP lysis buffer. The samples were mixed with an appropriate amount of 1x SDS loading buffer, boiled for 10 min, and then either frozen at −20 °C or immediately used for Western blot analysis.

### 2.12. Statistical Analysis

The trials were conducted three times, and the results are displayed as the mean ± SEM. Statistical significance was assessed by Student’s *t*-test to compare two groups, and *p* < 0.05 was deemed to be statistically significant.

## 3. Results

### 3.1. Bovine DDX3X Interacts with bSP110c through Its Helicase Domain

**Figure 1:**
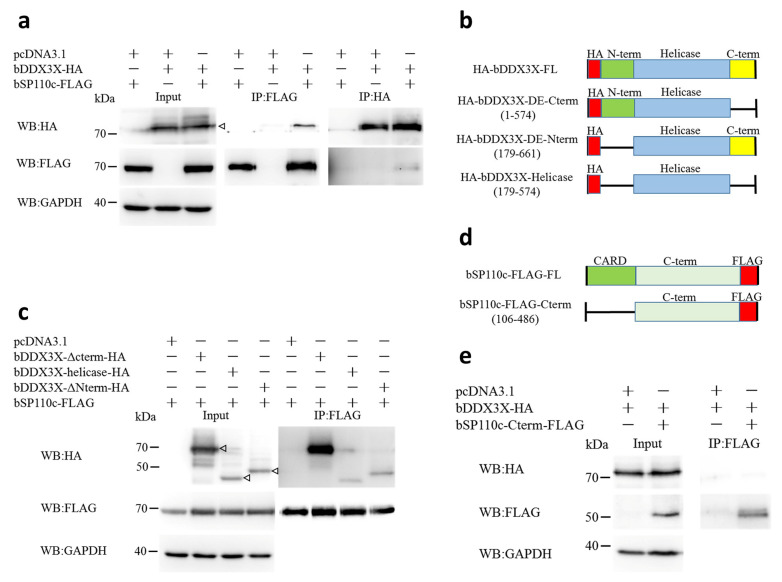
Bovine DDX3X interacts with bSP110c: (**a**) HEK293T cells were transfected with recombinant vectors for indicated expression of bDDX3X-HA and bSP110c-FLAG. Whole-cell extracts were co-IP with anti-Flag antibody or anti-HA antibody and analyzed by Western blot (WB) with respective antibodies. (**b**) Diagram of HA-tagged bDDX3X domain-deletion expression recombinant vectors. (**c**) bSP110c-FLAG and HA-labeled bDDX3X domain-deletion mutants were co-expressed in HEK293T cells. Whole-cell lysis underwent co-IP with anti-Flag antibody and was tested by WB with respective antibodies. (**d**) Diagram of FLAG-tagged bSP110c domain-deletion expression recombinant vectors. (**e**) bDDX3X-HA and Flag-labeled bSP110c domain-deletion mutants were co-expressed in HEK293T cells. Whole-cell lysis was subjected to co-IP with anti-Flag antibody and detected by WB with respective antibodies.

A total of 123 endogenous proteins interacting with bSP110c were identified using LC-MS/MS (Supplementary Table S1). We screened for functional proteins involved with inflammasome regulation and selected bDDX3X as a candidate protein for further investigation. In order to determine the interaction between bDDX3X and bSP110c, full-length and truncated versions of both proteins were co-transfected into HEK293T cells for co-IP analysis. The results indicated that bSP110c co-precipitated with bDDX3X (Figure 1a). Further examination of the interacting regions between the two proteins showed that bSP110c bound to the N-terminal and C-terminal shortened versions, along with the helicase domain of bDDX3X (Figure 1b,c). This indicated that the location where bSP110c binds is within the helicase region of bDDX3X. The protein structure of bSP110c shown in Figure 1d contains a CARD domain at its N-terminus. Notably, bDDX3X interacted with full-length bSP110c but not with its C-terminal region (Figure 1e). It was therefore possible that the interaction between bDDX3X and bSP110c might be mediated by the CARD domain of bSP110c.

### 3.2. Bovine DDX3X Suppresses bSP110c-Induced Inflammatory Response

**Figure 2:**
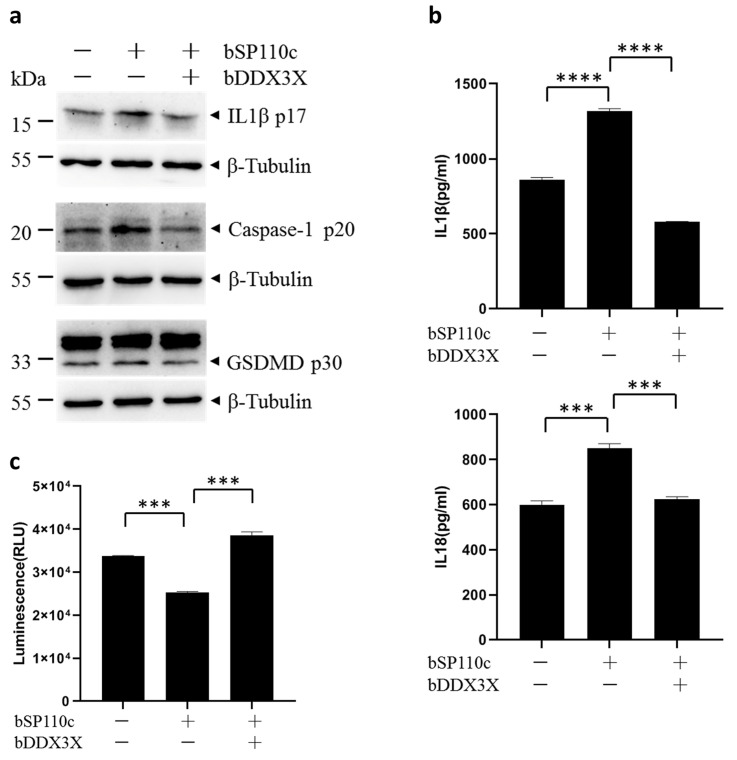
Bovine DDX3X suppresses bSP110c-induced inflammatory response: (**a**) WB of cleaved IL-1β, caspase-1, and GSDMD in whole-cell extracts of THP1 cells expressing bSP110c or co-expressing bSP110c and bDDX3X, primed with LPS. β-tubulin was also detected as loading control. (**b**) ELISA of IL-1β and IL18 in supernatants from LPS primed THP1 cells expressed of bSP110c or bSP110c and bDDX3X. **** *p* < 0.0001. (**c**) bSP110c expressed or bSP110c and bDDX3X co-expressed THP1 cells, treated with LPS were evaluated using an ATP-based luminescent cell viability assay. *** *p* < 0.0004.

Previous studies have demonstrated that bSP110c enhanced the release of IL-1β and IL-18 in THP1 macrophages infected with *L. monocytogenes* or treated with LPS. Hence, further inquiries were carried out to examine if bDDX3X controlled the inflammasome activation by bSP110c. THP1 cells expressing bSP110c or co-expressing bSP110c and bDDX3X (Figure S1a) were primed with LPS. bSP110c increased the activation of caspase-1 and pyroptosis indicator protein and gasdermin D (GSDMD), along with the processing of IL-1β (Figure 2a). Nevertheless, the trend was significantly reversed by co-expressing bDDX3X with bSP110c (Figure 2a). bSP110c greatly boosted the release of IL-1β and IL-18 in the culture supernatant, but this effect was strongly suppressed by bDDX3X (Figure 2b). Subsequently, bDDX3X also promoted cell survival upon bSP110c activation of inflammasomes (Figure 2c). This result was consistent with the changes in the cleaved GSDMD-N domain p30 (Figure 2a). These findings demonstrated that bDDX3X negatively regulated the bSP110c-mediated inflammatory response in stressed macrophages.

DDX3X was discovered as a protein associated with NLRP3 that controls the activation of NLRP3 inflammasomes in mouse macrophages [30]. We hypothesized that bDDX3X restricted the bSP110c-mediated inflammatory response via NLRP3 inflammasome.

### 3.3. Bovine DDX3X Regulates bSP110c Interaction with bNLRP3

**Figure 3:**
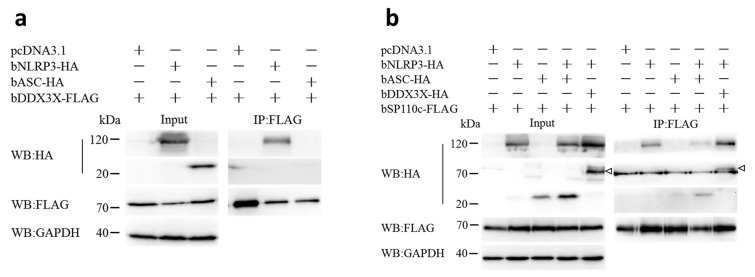
Bovine NLRP3 interacts with bDDX3X and bSP110c: (**a**) HEK293T cells were transfected with recombined constructs for indicated expression of bDDX3X-FLAG, bNLRP3-HA, and bASC-HA. Whole-cell extracts were subjected to co-IP with anti-Flag antibody or anti-HA antibody and detected by WB with respective antibodies. (**b**) HEK293T cells were transfected with recombined constructs for indicated expression of bSP110c-FLAG, bNLRP3-HA, bASC-HA, and bDDX3X-HA. Whole-cell extracts underwent co-IP with anti-Flag antibody or anti-HA antibody and were detected by WB with respective antibodies.

To investigate whether bDDX3X also interacted with the NLRP3 inflammasome, we co-expressed bDDX3X with bovine NLRP3 (bNLRP3) or bovine ASC (bASC) in HEK293T cells. Co-IP showed that bDDX3X co-precipitated with bNLRP3 but not with bASC (Figure 3a). This finding indicated that bDDX3X could potentially be a part of the NLRP3 inflammasome and play a role in controlling its function. To further study whether bDDX3X regulated the interaction of bSP110c with the NLRP3 inflammasome, we co-expressed bNLRP3 or bASC with bSP110c or both bSP110c and bDDX3X with bNLRP3 in HEK293T cells. Co-IP analysis demonstrated robust immunoprecipitation of bSP110c with bNLRP3 and a less pronounced immunoprecipitation with bASC (Figure 3b). Increased bNLRP3 expression promoted the connection between bASC and bSP110c (Figure 3b), indicating that bSP110c might be indirectly associated with bASC through bNLRP3. Moreover, increased levels of bDDX3X notably boosted the interaction between bSP110c and bNLRP3 in the co-precipitation assay (Figure 3b). These findings indicate that bDDX3X and bSP110c may integrate with bNLRP3 to form a dynamic molecular scaffold and regulate NLRP3 inflammasome activation.

### 3.4. Bovine DDX3X Inhibits NLRP3 Inflammasome Activation

**Figure 4:**
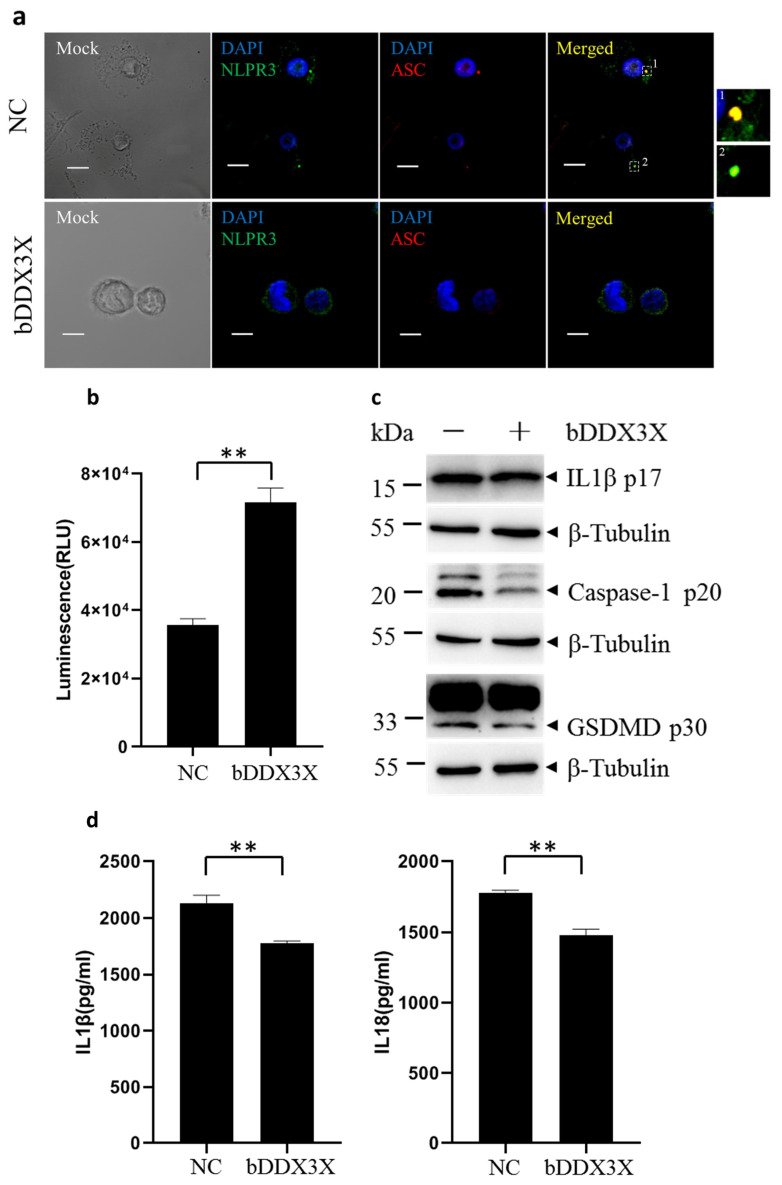
Bovine DDX3X inhibits pyroptosis and activation of NLRP3 inflammasomes in macrophages: (**a**) Confocal microscopy of THP1 cells expressing bDDX3X, pretreated with LPS, stimulated with nigericin, and stained for NLRP3 (green) ASC (red) and DAPI (blue). Scale bars, 10 μm. (**b**) THP1 cells expressing bDDX3X were treated with LPS and nigericin and evaluated using an ATP-based luminescent cell viability assay. ** *p* = 0.0014. (**c**) WB of cleaved IL-1β, caspase-1, and GSDMD in whole-cell extracts of bDDX3X-expressing THP1 cells, pretreated with LPS, and then stimulated with nigericin is shown. β-tubulin was also run as a loading control. (**d**) ELISA of IL-1β and IL18 in supernatants from THP1 cells expressing bDDX3X, incubated with LPS followed by nigericin ** *p* < 0.0078.

To further investigate the impact of bDDX3X on NLRP3 inflammasome activation, THP1 cells expressing bDDX3X were used (Figure S1b), and cells were exposed to LPS followed by nigericin to trigger the activation of the NLRP3 inflammasome [32]. Confocal microscopy showed that bDDX3X greatly decreased the typical pyroptosis morphology and formation of NLRP3 inflammasome-induced ASC specks in the cytoplasm (Figure 4a). This observation was consistent with the increased viability of THP1 cells expressing bDDX3X (Figure 4b). bDDX3X successfully decreased caspase-1 and GSDMD cleavage and promoted IL-1β maturation in the cytoplasm of THP1 cells stimulated by nigericin (Figure 4c). In comparison with the control group, the bDDX3X expression in THP1 cells led to a notable decrease in the release of IL-1β and IL-18 (Figure 4d). These findings indicate that bDDX3X has the ability to prevent pyroptosis and activation of the NLRP3 inflammasome in THP1 macrophages exposed to the NLRP3 inflammasome activator. These observations align with the idea that bDDX3X suppresses bSP110c-mediated caspase-1 cleavage, leading to reduced IL-1β and IL-18 generation. Thus, bDDX3X negatively regulates the bSP110c-induced inflammatory reaction by limiting NLRP3 inflammasome activation.

## 4. Discussion

As a vital part of innate immunity, much attention has been paid to the precise regulatory mechanism of inflammasome activation. IL-1 is crucial in the host immune reaction to infection [33,34]. However, the abnormal activation of the NLRP3 inflammasome due to mutations in the NLRP3 gene in humans has been linked to specific autoinflammatory syndromes and metabolic issues [35,36]. Therefore, in order to avert harmful inflammatory responses, stringent control mechanisms must be in place for inflammasomes.

Our laboratory identified variable splicing events in the transcriptome of the bovine *SP110* gene, resulting in the detection of three primary transcript variants. These expression products are designated as bSP110a isoform (GenBank accession no. AGT15801.1), bSP110b isoform (GenBank accession no. AGT15803.1), and bSP110c isoform (GenBank accession no. AGT15802.1). bSP110a is the full-length isoform, exhibiting high homology with human SP110c. The protein structure of bSP110b is consistent with that of mouse IPR1 and human SP110b [21]. The human homolog closest to the IPR1 protein is SP110b. By fine-tuning NF-κB activity, SP110b suppressed the production of TNF-α and enhanced the expression of antiapoptotic genes, thereby alleviating cell death caused by IFN-γ-mediated cytotoxicity and preventing the excessive amplification of these two cytokines [37,38]. SP110 was initially identified within PML (promyelocytic leukemia) nuclear bodies, which are multiprotein complexes located within the nucleus and involved in regulating various nuclear functions such as DNA replication, gene transcription, and epigenetic inheritance. Previous studies found high levels of *SP110* mRNA in human peripheral blood leukocytes and spleen, while it was undetectable in other tissues [39,40]. The SAND domain at the C-terminus of SP110 has been identified as a novel DNA-binding domain essential for regulating transcription. In vitro experiments have shown that human SP110 fused with the DNA-binding domain of yeast transcription factor GAL4 can significantly increase the activity of a CAT reporter system containing GAL4 binding sites [41,42].

Bovine SP110c has attracted much attention because of its specific protein structure. We compared the functional differences of bSP110c with bSP110a and bSP110b in regulating macrophage inflammatory responses in another study. We found that bSP110c enhances the activation of inflammasome, whereas bSP110a and bSP110b do the opposite. To further investigate the negative regulatory mechanism of the bSP110c–inflammasome signaling pathway, we focused on a potential negative regulator, DDX3X. Immunoprecipitation confirmed that bSP110c interacted with the bDDX3X helicase domain through its CARD domain. The co-expression of bDDX3X with bSP110c could certainly inhibit the activation of inflammasomes in LPS-treated THP1 macrophages, representing a novel mechanism for bDDX3X to negatively regulate the bSP110c-induced activation of inflammasomes.

DDX3X was initially recognized for its involvement in the translation of both host and viral RNA [43,44]. As a part of the DEAD-box family, DDX3X contains a helicase core that is highly conserved. Bovine DDX3X shares 99.1 percent identity with human DDX3X. Both the human and murine *DDX3X* genes have shown the capacity to compensate for the lack of the *Ded1* yeast ortholog [45,46]. DDX3X acts as a regulator of IFN-1 transcription in cells infected with viruses or the intracellular bacterial pathogen *L. monocytogenes* [47,48]. It has also been reported to interact with various binding partners, such as TBK1, IKBKE, CHUK, and NLRP3 [49,50,51,52]. This study confirmed that bDDX3X interacted with bSP110c and enhanced the interaction between bSP110c and bNLRP3. The new connection between bDDX3X and bSP110c, forming a complex with bNLRP3, as reported here, has unveiled a comprehensive landscape of DDX3X interacting proteins. Even so, the dynamics of bDDX3X and bSP110c recruitment to NLPR3 during inflammasome activation need further research.

**Figure 5:**
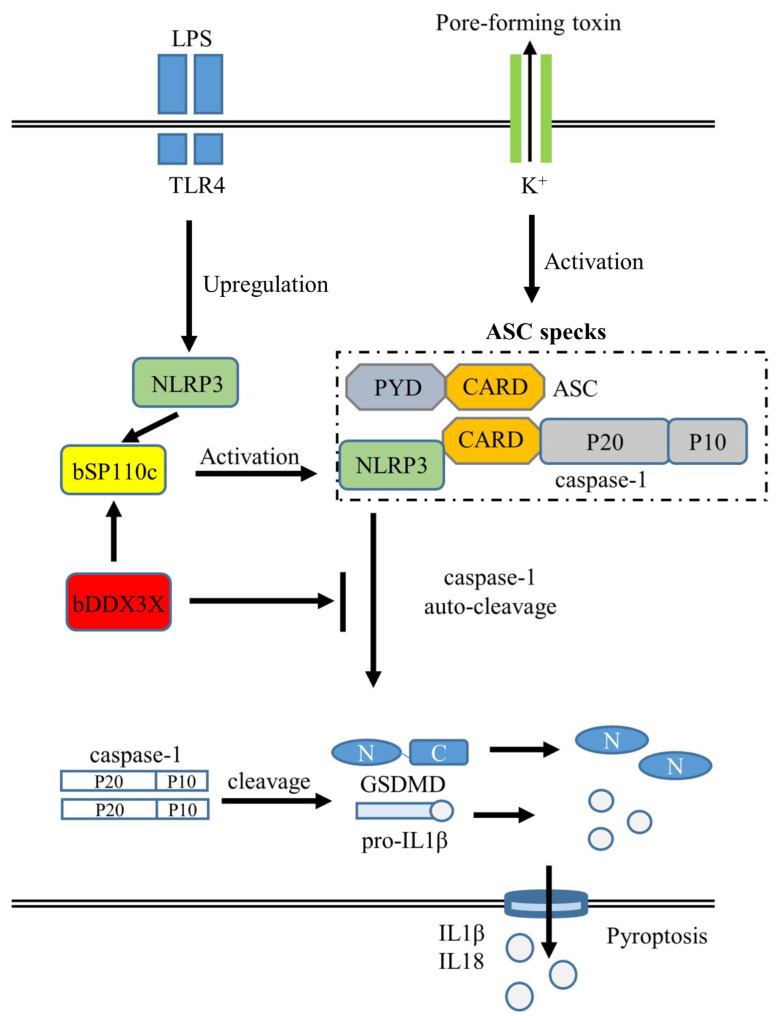
Diagram of the effects of interaction between bDDX3X and bSP110c and its relationship with inflammasome activation and cell-fate decisions. bDDX3X inhibits NLRP3 inflammasome activation probably by interacting with NLRP3. bDDX3X restrains bSP110c activation of NLRP3 inflammasomes in macrophages most likely by interacting with the bSP110c CARD domain through its helicase domain and regulating bSP110c interaction with NLRP3.

Multiple pieces of evidence indicate that bDDX3X functions as a suppressor of NLRP3 inflammasomes: (a) bDDX3X decreases the production of ASC specks and pyroptosis upon NLRP3 inflammasome activation in THP-1 cells; (b) bDDX3X interacts with bNLRP3 but not with bASC; and (c) the cleavage of caspase-1 and GSDMD mediated by NLRP3 inflammasomes and the generation of IL-1β and IL-18 are notably suppressed by bDDX3X. By observing comparable inhibitions of the inflammasome, we verified that bDDX3X hinders the bSP110c-induced inflammatory reaction by negatively regulating the NLRP3 inflammasome (Figure 5). We speculate that there may be a negative feedback mechanism involving bDDX3X-bSP110c that regulates the activation of inflammasomes in vivo. Through the precise activation of negative regulators, the host immune system strictly controls the production of proinflammatory factors to avoid pernicious and inappropriate inflammatory responses. This regulation pattern needs further investigation.

## 5. Conclusions

Overall, the findings here demonstrate that bDDX3X is a novel bSP110c binding protein and provide evidence of a new cooperative mechanism between bSP110c and bDDX3X to balance inflammasome activation. These discoveries advance our understanding of the special genetic factor, SP110c, in the bovine innate immunity system, providing a theoretical basis for further application in developing disease-resistant cattle through biotechnological breeding.

## Data Availability

The data presented in this study are available upon reasonable request from the corresponding author.

## Supplementary Materials

The following supporting information can be downloaded at: https://www.mdpi.com/article/10.3390/ani14111650/s1, Figure S1: Identification of bovine protein expression. Table S1: Bovine SP110c protein interactome identified by mass spectrometric.
