# Supplementary material for: Bovine DDX3X Restrains Bovine SP110c-Mediated Activation of Inflammasome in Macrophages
Source: Animals (Basel). 2024 May 31;14(11):1650. doi: 10.3390/ani14111650 (PMC11171048; doi:10.3390/ani14111650)
Supplement: Supplementary file 1 [file animals-14-01650-s001.zip › Figure S1.pdf]

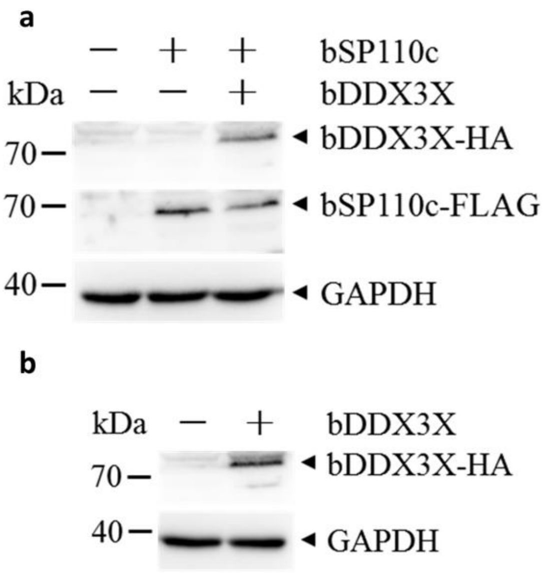

Figure S1. Identification of bovine protein expression. (**a**) Immunoblot of bSP110c expression and bDDX3X and bSP110c co-expression in THP1 cells. (**b**) Immunoblot of bDDX3X expression in THP1 cells. The GAPDH was also detected as loading control.
