# Supplementary figures and images for: Bovine DDX3X Restrains Bovine SP110c-Mediated Activation of Inflammasome in Macrophages
Source: Animals (Basel). 2024 May 31;14(11):1650. doi: 10.3390/ani14111650 (PMC11171048; doi:10.3390/ani14111650)

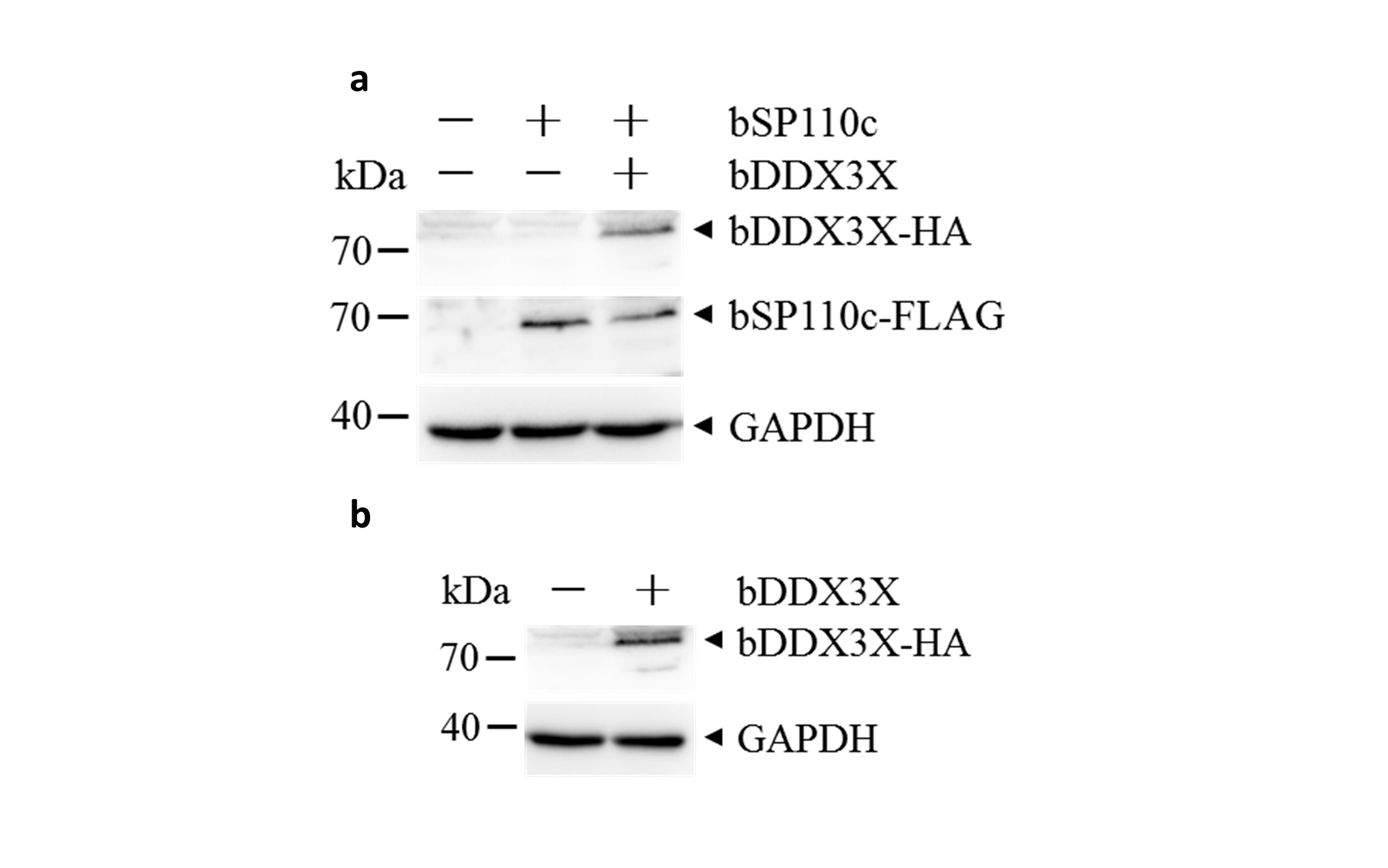

Supplement: Supplementary file 1 [file animals-14-01650-s001.zip › Extended Data Fig S1.tif]
